# Supplementary material for: Loss of immune tolerance to IL-2 in type 1 diabetes
Source: Nat Commun. 2016 Oct 6;7:13027. doi: 10.1038/ncomms13027 (PMC5059699; doi:10.1038/ncomms13027)
Supplement: Supplementary Information — Supplementary Figures 1-4, Supplementary Tables 1-4 and Supplementary Methods [file ncomms13027-s1.pdf]

Supplementary figures:

Supplementary Fig 1

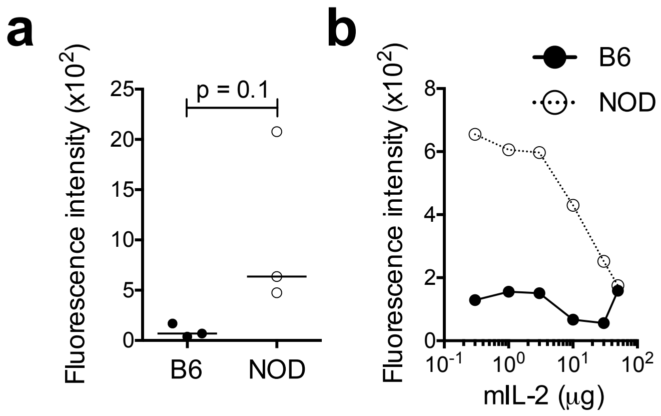

Fig. S1. Control of anti-mIL-2 autoantibodies specificity.

(a) Titers of anti-murine-IL-2 IgG were quantified by FACS with IL-2 coated fluorescent beads. (b) Competition assay: sera from an anti-mIL-2 negative B6 mouse (closed circles) or from an anti-mIL-2 positive pre-diabetic NOD mouse (open circles) were pre-incubated for 1h with increasing amounts of free recombinant mIL-2 and titers of anti-mIL-2 were then quantified by FACS with IL-2 coated fluorescent beads. Symbols represent individual mice and horizontal bars are the medians. Data are cumulative of at least two independent experiments.

Supplementary figure 2

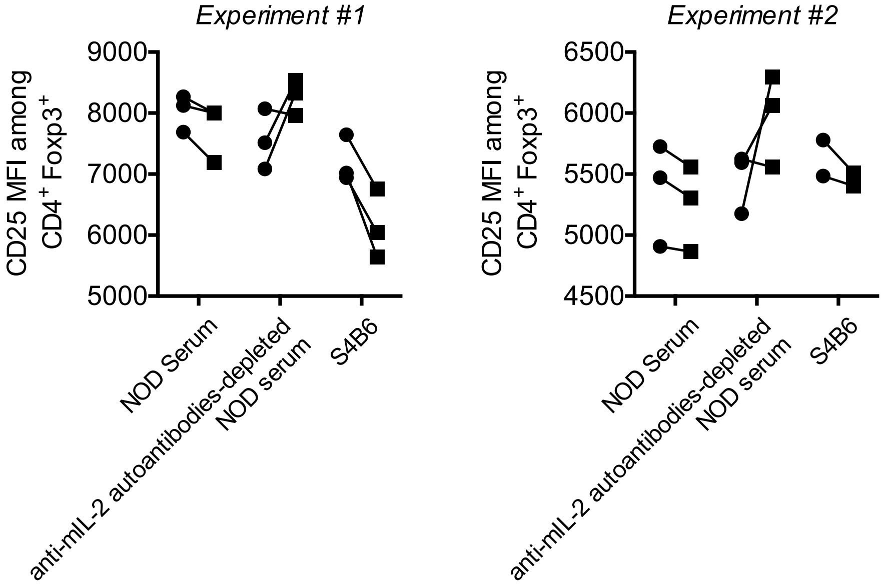

Fig. S2. Effect of anti-mIL-2 autoantibodies on T_{reg} cell homeostasis.

In two independent experiments (#1 and #2), male NOD mice of 12 weeks of age were daily injected for 2 days with total NOD serum (*n*=3), anti-mIL-2-autoantibodies depleted NOD serum (*n*=3) or anti-IL-2 (clone S4B6, 300 ng/injection, *n*=3 in experiment #1, *n*=2 in experiment #2). Quantification of CD25 MFI among T_{reg} cells in the blood of mice of the indicated groups. Shown are values obtained in individual mice at day 0, before serum transfer and at day 2, 24 h after the second serum transfer in the two independent experiments. Symbols represent individual mice.

Supplementary Figure 3

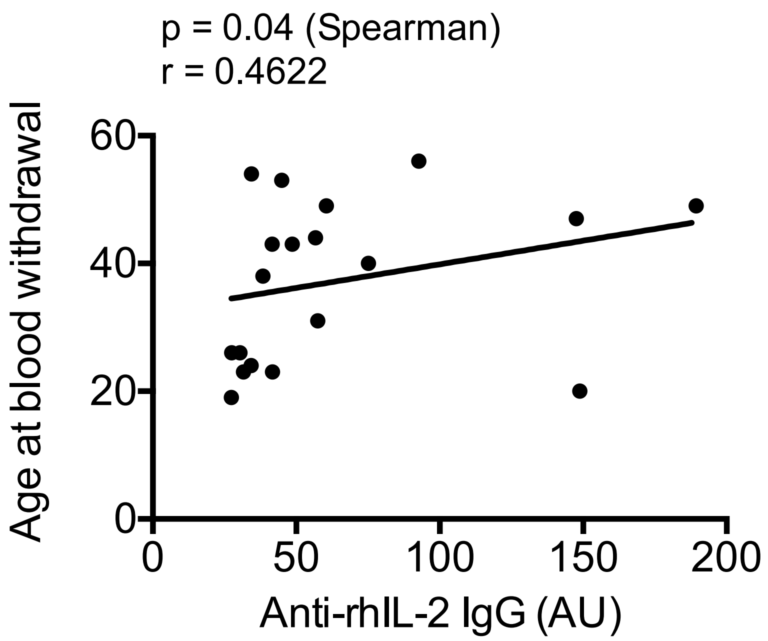

Fig. S3. Anti-rhIL-2 autoantibody titers correlate with age in T1D patients.

Serum samples were obtained from T1D patients and titers of anti-rhIL-2 IgG were determined by ELISA as described above. Correlation between anti-rhIL-2 IgG titers and age at blood withdrawal in the anti-rhIL-2$^+$ T1D patients (non-parametric Spearman correlation test).

Supplementary Figure 4

```
murine IL-2 MYSMQLASCVTLTLVLLVNSAPTSSSTSSSTAEAQQQQQQQQQQQQHLEQLLMDLQELLSRMENYRNLK
            || ||| ||    | |  |||||||||                    || || |||  |    || | |
 human IL-2 MYRMQLLSCIALSLALVTNSAPTSSSTKKTQLQ--------------LEHLLLDLQMILNGINNYKNPK

murine IL-2 LPRMLTFKFYLPKQATELKDLQCLEDELGPLRHVLDLTQSKSFQLEDAENFISNIRVTVVKLKGSDNTF
            | |||||||| || ||||| ||||| || ||  || | ||| | |      |||| | |  ||||  ||
 human IL-2 LTRMLTFKFYMPKKATELKHLQCLEEELKPLEEVLNLAQSKNFHLR-PRDLISNINVIVLELKGSETTF

murine IL-2 ECQFDDESATVVDFLRRWIAFCQSIISTSPQ
             |   || || | || ||| ||||||||
 human IL-2 MCEYADETATIVEFLNRWITFCQSIISTLT
```

Fig. S4. Sequence alignment of mIL-2 and rhIL-2.

The amino acid sequences of mIL-2 and rhIL-2 were aligned with NCBI's BLAST. The immunodominant peptides of mIL-2 and rhIL-2 are in bold.

Supplementary tables:

Supplementary Table 1 – Murine IL-2 peptide library

Peptide ID	Sequence
mIL-2$_{1-15}$	MYSMQLASCVTLTLV
mIL-2$_{4-18}$	MQLASCVTLTLVLLV
mIL-2$_{7-21}$	ASCVTLTLVLLVNSA
mIL-2$_{10-24}$	VTLTLVLLVNSAPTS
mIL-2$_{13-27}$	TLVLLVNSAPTSSST
mIL-2$_{16-30}$	LLVNSAPTSSSTSSS
mIL-2$_{19-33}$	NSAPTSSSTSSSTAE
mIL-2$_{22-36}$	PTSSSTSSSTAEAQQ
mIL-2$_{25-39}$	SSTSSSTAEAQQQQQ
mIL-2$_{28-42}$	SSSTAEAQQQQQQQQ
mIL-2$_{31-45}$	TAEAQQQQQQQQQQQ
mIL-2$_{34-48}$	AQQQQQQQQQQQQHL
mIL-2$_{37-51}$	QQQQQQQQQQHLEQL
mIL-2$_{40-54}$	QQQQQQQHLEQLLMD
mIL-2$_{43-57}$	QQQQHLEQLLMDLQE
mIL-2$_{46-60}$	QHLEQLLMDLQELLS
mIL-2$_{49-63}$	EQLLMDLQELLSRME
mIL-2$_{52-66}$	LMDLQELLSRMENYR
mIL-2$_{55-69}$	LQELLSRMENYRNLK
mIL-2$_{58-72}$	LLSRMENYRNLKLPR
mIL-2$_{61-75}$	RMENYRNLKLPRMLT
mIL-2$_{64-78}$	NYRNLKLPRMLTFKF
mIL-2$_{67-81}$	NLKLPRMLTFKFYLP
mIL-2$_{70-84}$	LPRMLTFKFYLPKQA
mIL-2$_{73-87}$	MLTFKFYLPKQATEL
mIL-2$_{76-90}$	FKFYLPKQATELKDL
mIL-2$_{79-93}$	YLPKQATELKDLQCL
mIL-2$_{82-96}$	KQATELKDLQCLEDE
mIL-2$_{85-99}$	TELKDLQCLEDELGP
mIL-2$_{88-102}$	KDLQCLEDELGPLRH
mIL-2$_{91-105}$	QCLEDELGPLRHVLD
mIL-2$_{94-108}$	EDELGPLRHVLDLTQ
mIL-2$_{97-111}$	LGPLRHVLDLTQSKS
mIL-2$_{100-114}$	LRHVLDLTQSKSFQL
mIL-2$_{103-117}$	VLDLTQSKSFQLEDA
mIL-2$_{106-120}$	LTQSKSFQLEDAENF
mIL-2$_{109-123}$	SKSFQLEDAENFISN
mIL-2$_{112-126}$	FQLEDAENFISNIRV
mIL-2$_{115-129}$	EDAENFISNIRVTVV
mIL-2$_{118-132}$	ENFISNIRVTVVKLK
mIL-2$_{121-135}$	ISNIRVTVVKLKGSD
mIL-2$_{124-138}$	IRVTVVKLKGSDNTF
mIL-2$_{127-141}$	TVVKLKGSDNTFECQ
mIL-2$_{130-144}$	KLKGSDNTFECQFDD
mIL-2$_{133-147}$	GSDNTFECQFDDESA
mIL-2$_{136-150}$	NTFECQFDDESATVV
mIL-2$_{139-153}$	ECQFDDESATVVDFL
mIL-2$_{142-156}$	FDDESATVVDFLRRW
mIL-2$_{145-159}$	ESATVVDFLRRWIAF
mIL-2$_{148-162}$	TVVDFLRRWIAFCQS
mIL-2$_{151-165}$	DFLRRWIAFCQSIIS
mIL-2$_{154-168}$	RRWIAFCQSIISTSP
mIL-2$_{157-169}$	IAFCQSIISTSPQ

Supplementary Table 2 – Demographic and clinical characteristics of the T1D cohorts

	T1D Cohort 1 (Paris, n=39)	T1D Cohort 2 (DASP, n=15)	T1D Cohort 3 (Italy, n=21)
Sex, no. (%) female	19 (48.7)	8 (53.3)	12 (57.1)
Age at T1D onset, median (range), years	23 (3, 55)	26 (18, 37)	28 (7, 43)
Age at sample collection, median (range), years	42 (20, 73)	26 (18, 37)	39 (20, 56)
T1D status at sample collection			
< 1 week after diagnosis, no. (%)	0 (0)	15 (100)	8 (38.1)
> 1 week after diagnosis, no. (%)	39 (100)	0 (0)	13 (61.9)
Interval between diagnosis and sample collection, median (range), years	19 (0.5, 47)	NA	12 (0.3-35)

NA : Not applicable

Supplementary Table 3 – Demographic and clinical characteristics of the T2D cohort

	T2D Cohort (n=24)
Sex, no. (%) female	10 (41.7)
Age at T2D onset, median (range), years	49 (29, 64)
Age at sample collection, median (range), years	60 (41, 73)
Body mass index (BMI), median (range), kg/m^2	31.1 (18.6, 44)

Supplementary Table 4 – Human IL-2 peptide library

Peptide ID	Sequence
rhIL-2_{1-15}	MYRMQLLSCIALSLA
rhIL-2_{6-20}	LLSCIALSLALVTNS
rhIL-2_{11-25}	ALSLALVTNSAPTSS
rhIL-2_{16-30}	LVTNSAPTSSSTKKT
rhIL-2_{21-35}	APTSSSTKKTQLQLE
Pro_{1-15}	MPTSSSTKKTQLQLE
rhIL-2_{26-40}	STKKTQLQLEHLLLD
rhIL-2_{31-45}	QLQLEHLLLDLQMIL
rhIL-2_{36-50}	HLLLDLQMILNGINN
rhIL-2_{41-55}	LQMILNGINNYKNPK
rhIL-2_{46-60}	NGINNYKNPKLTRML
rhIL-2_{51-65}	YKNPKLTRMLTFKFY
rhIL-2_{56-70}	LTRMLTFKFYMPKKA
rhIL-2_{61-75}	TFKFYMPKKATELKH
rhIL-2_{66-80}	MPKKATELKHLQCLE
rhIL-2_{71-85}	TELKHLQCLEEELKP
rhIL-2_{76-90}	LQCLEEELKPLEEVL
rhIL-2_{81-95}	EELKPLEEVLNLAQS

Peptide ID	Sequence
rhIL-2_{86-100}	LEEVLNLAQSKNFHL
rhIL-2_{91-105}	NLAQSKNFHLRPRDL
rhIL-2_{96-110}	KNFHLRPRDLISNIN
rhIL-$2_{101-115}$	RPRDLISNINVIVLE
rhIL-$2_{106-120}$	ISNINVIVLELKGSE
rhIL-$2_{111-125}$	VIVLELKGSETTFMC
rhIL-$2_{116-130}$	LKGSETTFMCEYADE
rhIL-$2_{121-135}$	TTFMCEYADETATIV
rhIL-$2_{126-140}$	EYADETATIVEFLNR
rhIL-$2_{131-145}$	TATIVEFLNRWITFC
$Pro_{111-125}$	TATIVEFLNRWITFS
rhIL-$2_{136-150}$	EFLNRWITFCQSIIS
$Pro_{116-130}$	EFLNRWITFSQSIIS
rhIL-$2_{141-153}$	WITFCQSIISTLT
$Pro_{121-133}$	WITFSQSIISTLT
rhIL-$2_{139-153}$	NRWITFCQSIISTLT
$Pro_{129-133}$	NRWITFSQSIISTLT

Supplementary Methods:

Multiplex particle-based flow cytometry

Recombinant mIL-2 was covalently coupled to carboxylated beads (Bio-Rad Laboratories). Beads were first activated with 1-ethyl-3-[3-dimethylaminopropyl]carbodiimide hydrochloride in the presence of N-hydroxysuccinimide (Thermo Fisher), according to the manufacturer's instructions, to form amine-reactive intermediates. The activated beads were incubated with 10 µg/mL mIL-2 in the reaction mixture for 2 h at room temperature under rotation. Beads were then blocked and stored according to the manufacturer's instructions. Coupling was verified using a commercial anti-mIL-2 monoclonal antibody (clone JES6-1A12, eBioscience), biotin-anti-rat Ig (BD Biosciences) and then PE-streptavidin (Invitrogen). mIL-2-coupled beads were incubated with serially diluted sera from B6 or NOD mice for 2 h in 96-well plates at room temperature in the dark on a horizontal shaker. Beads were washed twice with PBS/0.05% Tween-20 and incubated for 1 h with a biotin-labeled anti–murine IgG antibody (1:250; Southern Biotech), washed, incubated 30 min with PE-streptavidin (1:125; Invitrogen), washed again and resuspended in 100 µL PBS/0.05% Tween-20. Beads were then analyzed on LSRII flow cytometer (BD Biosciences) and data analyzed with FlowJo software. For anti-mIL-2 autoantibodies competition assays, sera from B6 or NOD mice (diluted 1/10) were pre-incubated with increasing concentrations of mIL-2 for 2 h at room temperature. mIL-2 coated beads were then added and multiplex particle-based flow cytometry processed as described above.

Serum transfer experiment

Serum was collected from anti-mIL-2-autoantibodies ^{high} NOD mice and was pooled. One half of the pooled serum was incubated in mIL-2-coated columns (50 µg mIL-2 per column, Microlink Protein Kit, Thermo Scientific) overnight at 4°C with end-over-end mixing. The mIL-2A-depleted fraction was recovered by centrifugation and was submitted to another round of depletion. This procedure led to a 60-fold reduction of the mIL-2A IgG titers, as determined by ELISA (see below for the ELISA protocol). As a positive control, we used an anti-IL-2 monoclonal antibody (clone S4B6, BD Biosciences) at a concentration equivalent to the one estimated in the pooled NOD serum. The concentration of mIL-2A in the pooled serum was estimated by ELISA (see above for the ELISA protocol) using plates coated with mIL-2 (1 µg/mL) or rhIL-2 (1 µg/mL) and using serially diluted murine anti-human IL-2 IgG (BD Biosciences, clone 5344.111) as a standard. We then daily injected i.p. 12-week-old male

NOD mice with 100 μL of total serum, mIL-2A-depleted serum or S4B6 (3 μg/mL) during 2 days. Flow cytometry was performed on day 0 and day 2.
